# Remote photoplethysmography (rPPG) in the wild: Remote heart rate imaging via online webcams

**DOI:** 10.3758/s13428-024-02398-0

**Published:** 2024-04-17

**Authors:** Daniele Di Lernia, Gianluca Finotti, Manos Tsakiris, Giuseppe Riva, Marnix Naber

**Affiliations:** 1 Humane Technology Lab, Università Cattolica del Sacro Cuore, Largo Gemelli, 1, 20100 Milan, Italy; 2 Lab of Action and Body, Department of Psychology, Royal Holloway, University of London, Egham Hill, Egham, TW20 0EX UK; 3 Centre for the Politics of Feelings, School of Advanced Study, University of London, London, UK; 4 Applied Technology for Neuro-Psychology Lab, IRCCS Istituto Auxologico Italiano, Via Magnasco, 2, 20149 Milan, Italy; 5 Experimental Psychology, Helmholtz Institute, Utrecht University, Heidelberglaan 1, 3584CS Utrecht, The Netherlands; 6 Department of Psychology, Università Cattolica del Sacro Cuore, Largo Gemelli, 1, 20100 Milan, Italy

**Keywords:** Remote photoplethysmography, Heart rate, Interoception, Interoceptive perception

## Abstract

Remote photoplethysmography (rPPG) is a low-cost technique to measure physiological parameters such as heart rate by analyzing videos of a person. There has been growing attention to this technique due to the increased possibilities and demand for running psychological experiments on online platforms. Technological advancements in commercially available cameras and video processing algorithms have led to significant progress in this field. However, despite these advancements, past research indicates that suboptimal video recording conditions can severely compromise the accuracy of rPPG. In this study, we aimed to develop an open-source rPPG methodology and test its performance on videos collected via an online platform, without control of the hardware of the participants and the contextual variables, such as illumination, distance, and motion. Across two experiments, we compared the results of the rPPG extraction methodology to a validated dataset used for rPPG testing. Furthermore, we then collected 231 online video recordings and compared the results of the rPPG extraction to finger pulse oximeter data acquired with a validated mobile heart rate application. Results indicated that the rPPG algorithm was highly accurate, showing a significant degree of convergence with both datasets thus providing an improved tool for recording and analyzing heart rate in online experiments.

## Introduction

Remote photoplethysmography (rPPG) is a method that utilizes video recordings to collect physiological parameters – such as heart rate, respiration rate, and oxygenation – remotely through light variations on the skin surface (van der Kooij and Naber 2019). rPPG is based on the concept that heartbeat pulsations produce changes in skin blood perfusion and that such changes can be measured through variations in luminosity (Hertzman, 1937). Using this principle, Verkruysse, Svaasand et al. (2008) demonstrated that heart rate measurements could also be achieved through the remote detection of normal light variations on the skin with consumer cameras. In this case, rPPG works via a process called photo-amplification, or Eulerian video magnification, able to remotely detect and enhance variations in the reflected colors of the skin that are caused by changes in capillary tissue movement (Wu et al., 2012, Kamshilin, Nippolainen et al. 2015).

Several open-source implementations of this technology exist and are freely available in code repositories (McDuff and Blackford 2019; van der Kooij and Naber 2019; Boccignone et al., 2020). However, rPPG’s accuracy depends on specific setup requirements, including camera specifications, illumination, video encoding, facial movements, and the general setting (e.g., a constant neutral background). Suboptimal conditions may severely impair the possibility of using rPPG techniques in out-of-lab environments, such as during online data collection, where researchers cannot control the setting beforehand, the illumination, or the hardware used by the participants. While several video databases for rPPG benchmarking are openly available (Soleymani, Lichtenauer et al. 2012, Stricker et al., 2014, Heusch et al., 2017, Bobbia, Macwan et al. 2019), finding a way to include rPPG in online experiments could provide researchers with a tool that can be deployed to collect physiological parameters remotely, fostering large-scale psychophysiological data collections outside the lab.

Since several initial studies, subsequent research has concentrated on increasing rPPG resilience to various types of interference, such as video noise, participant movement, and lighting conditions (for reviews, see (McDuff, Estepp et al. 2015, Sun and Thakor 2016, Rouast, Adam et al. 2018, Sinhal et al., 2020). Nonetheless, thus far, no extant literature has empirically validated the efficacy of rPPG algorithms under arguably one of the most challenging data acquisition conditions – namely, video recordings self-generated by participants via web browsers in environments devoid of experimental control. While a recent study utilized the rPPG methodology described in this paper to analyze webcam videos collected online, identifying a relationship between interoceptive heart rate detection abilities and political preferences (Ruisch, Mohr et al. 2023), it remains unclear whether rPPG has the sensitivity to accurately detect heart rate from low-quality videos recorded using pre-set JavaScript-based browser software.

For these reasons, we set out to test a modified rPPG algorithm along with a collection and pre-processing pipeline able to extract rPPG data from noisy videos recorded via an online platform. The solution presented tests suboptimal conditions that usually impair common rPPG algorithms’ ability to extract reliable data when illumination, framing, background, and movements are not controlled for. Moreover, the presented solution can work with basic software requirements, needing only a web browser, without the necessity to control participants’ hardware (e.g., PC and webcam), and with very short video recordings (starting from 25 s) if minimum frames per seconds parameters are met. Taken together, these improvements constitute a very flexible and powerful tool that can easily be deployed in online data collections, allowing researchers to record and utilize physiological measures in large-scale online experiments and psychophysiological paradigms.

In two studies, we will demonstrate rPPG extraction merged with an online experimental platform that allows the recording of participants’ heart rate despite the limited control over the context and the setup. In study 1, we first compared the results of a modified open-source rPPG extraction algorithm (van der Kooij and Naber 2019) to a validated dataset (CohFace) used for rPPG testing, assessing the convergence of the rPPG algorithm results versus blood-volume pulse (BVP) data collected directly with a sensor from Thought Technologies (for further details see Heusch et al. (2017)). In the second study, we collected 231 online video recordings of 18 participants and we compared the results of the rPPG extraction against finger pulse oximeter data, collected with a validated mobile heart rate application (Losa-Iglesias, Becerro-de-Bengoa-Vallejo et al. 2016).

## Methods

### Study 1: CohFace dataset

#### Stimuli

We tested the rPPG algorithm on the CohFace video dataset (Heusch, Anjos et al. 2017) because the video properties and light conditions most closely match those of the videos recorded in Study 2. The CohFace dataset includes 164 videos from 40 individuals. The average subject age is 35.6 years old, with a standard deviation of 11.47 years. The gender of the participants was 12 women (30%) and 28 men (70%). Each face is recorded with a Logitech HD Webcam C525, at a resolution of 640 × 480 and a frame rate of 20 FPS, for a duration of 60 s. At the same time, blood-volume pulse (BVP) and respiration were also recorded, with a sensor from Thought Technologies (BBVP model SA 9308M); for more information, see (Heusch, Anjos et al. 2017). Moreover, each participant was recorded in two different lighting conditions: (1) studio quality (that is, closed blinds, minimizing natural light, with extra light from a spot to keep the face of the subject well illuminated); (2) with natural light (artificial lights were turned off and the blinds were opened).

#### Analysis

As a first step, we extracted the real number of heartbeats from the BVP trace. To this aim, Heusch and Marcel report using a simple peak-detector available as free software. However, visual inspection showed that several BVP traces presented slow-frequency and/or high-frequency noise. For this reason, first, we normalized the data by applying the MATLAB *normalize* function. BVP signals were then detrended and filtered with the *smoothdata* function using a moving window (set at 50). Only then did we use the *findpeaks* function, with a minimum peak distance of 450 ms. Visual inspection showed that this procedure was effective in reliably detecting BVP peaks in most cases with the exception of 12 BVP traces. These, and the relative videos, were excluded from further analysis resulting in a final sample of *N* = 152 videos.

#### Data cleaning

All the videos were processed with our rPPG algorithm. As a first step, we removed videos for which the rPPG HR could not be computed because the computer vision toolbox could not recognize a face (ten videos removed), possibly due to a horizontal luminance gradient caused by light coming from the side. All videos had a frame rate higher than 19.9, which, as a rule of thumb, and based on previous experiences, is the absolute minimum for rPPG. Therefore, no video had to be removed due to a low frame rate. We removed all videos for which the real HR (0 videos) or the rPPG HR (one video) was lower than 50 or higher than 120 beats per minute (BPM). Finally, we used the r *boxplot* function to detect and remove outliers, defined as the values outside the interquartile range, both for the Real HR and the rPPG HR. No outliers were found with this method. This process left us with a total of 141 videos from 39 different participants for the final analysis.

#### rPPG extraction

**Fig. 1:**
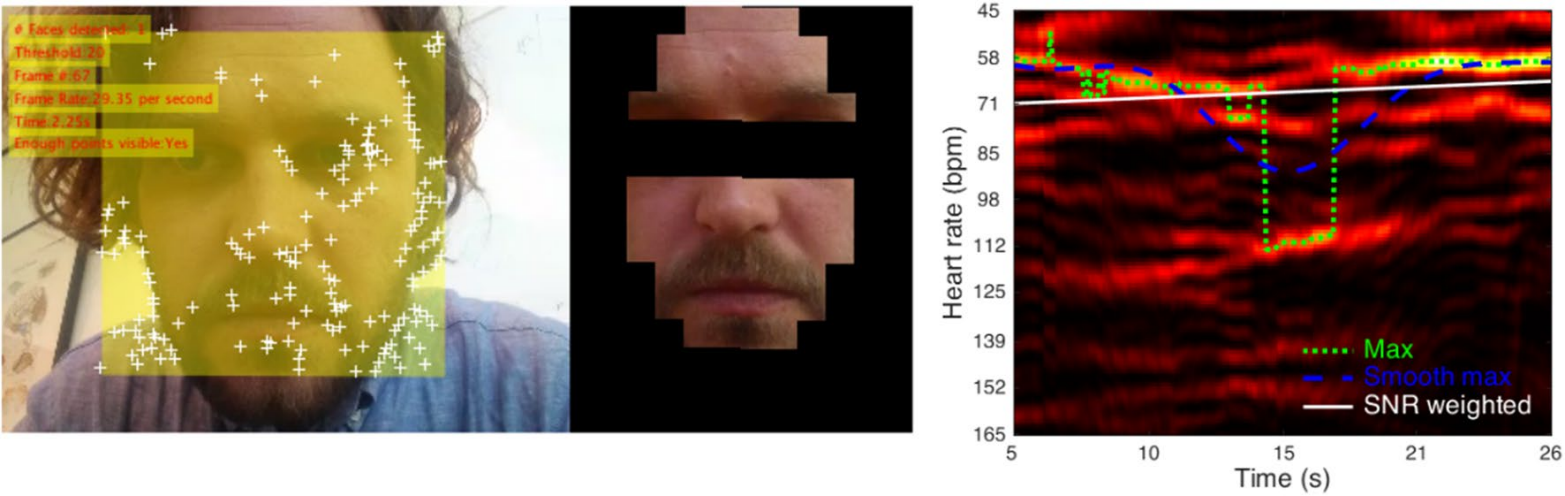
On the left, a snapshot of the RPPG algorithm’s detection of the face location (*yellow square*), unique features to track head rotations (*white crosses*), and a skin mask placed on top of the face depending on head rotations. It shows, on the right, the results of a time–frequency analysis with time in seconds on the *x*-axis and heart rate in beats per minute on the *y*-axis. *Brighter colors* indicate higher signal power. The *overlain time traces* indicate functions representing heart rate based on maximum power values of a raw (*green*, *dotted*) or smoothed (*blue*, *dashed*) function, or based on SNR-weighted power values (*white*, *solid*). All results presented in this work are based on the heart rate estimated by the smoothed function

Analyses of the videos were conducted in MATLAB. Most analysis steps are described in detail by van der Kooij and Naber (2019) but some modifications were made to improve the accuracy of the rPPG algorithm or to speed up the analysis process. Figure 1 highlights the most relevant analysis steps of the rPPG algorithm. The first step consists of the detection of the face in the first frame using the computer vision toolbox cascade object detector that uses a Viola–Jones algorithm trained on the FrontalFaceLBP dataset (Lienhart, Kuranov et al. 2003). As face detection is a computationally demanding procedure, we decided to efficiently track features within the face rather than redetecting the face in each frame. As such, we detected unique feature points in the face that consisted of corner points as detected with a minimum eigenvalue algorithm (Jianbo Shi and Tomasi 1994). These features were tracked in the following frames using the computer vision toolbox point tracker that uses the Kanade–Lucas–Tomasi algorithm (KLT; (Lucas and Kanade 1981; Tomasi and Kanade 1991). In contrast to the relatively slow color-based skin detection method described in van der Kooij and Naber (2019), we used a rough template of a face to more swiftly localize skin areas above and below the eyes. The template was adjusted in size and rotated in 2D space depending on the spatial orientation of the tracked features per frame. The average RGB values across all skin pixels was stored per frame, resulting in a 3 (RGB color channels) by *n* (number of frames) matrix as input for the rPPG analysis. To roughly equalize data power and ease comparison across subjects, which depends on the frame rate and differed across videos, this array was resampled to 60 Hz (maximum possible frame rate in the online videos and CohFace dataset) using MATLAB’s pchip interpolation method for all videos. Next, each color channel was band-pass filtered using a Butterworth filter (0.75–2.75 Hz; 6th order). We then dimension-reduced the RGB array, initially representing changes in average 3D RGB color space, to a single array, eventually representing only heart-beat-related fluctuations, using the plane-orthogonal-to-skin algorithm (POS; Wang, Li et al. 2022)) with a sliding window size of 1.6 s. This final array was converted to the frequency domain using a time–frequency analysis based on Lomb–Scargle periodogram calculations per sliding window of 10 s with a temporal resolution of 240 points and frequency resolution of 120 points. The resulting power density functions, represented in a 2D (240 by 120) data array, were converted to signal-to-noise ratios (i.e., SNR; also termed coherence) per time point by dividing each power value by the sum of all absolute power values. Only the frequencies with the 5th percentile largest SNR values were selected to calculate heart rate. The final rPPG HR measure was based on the SNR-weighted average of frequencies. In noisy videos, the frequency with the strongest power in the time-frequency analysis could vary suddenly from frame to frame. As such, we applied a smoothed fit to the peak powers across time to reduce distortions by spurious changes in frequencies with high powers.

#### Accuracy

First, we checked whether our variables of interest were normally distributed by visual inspection of Quantile-Quantile Plots (Q-Q plots, which compare two probability distributions by plotting their quantiles against each other), Skewness (that is, the symmetry of the data) and Kurtosis (that is, whether the data are heavy-tailed, or light-tailed). Furthermore, we performed two separate Shapiro–Wilk tests of normality, one for True HR and one for rPPG HR. Because data were not normally distributed, we used non-parametric tests.

To test the convergence of the HR as measured with the rPPG, we compared the rPPG HR to the ground truth (i.e., BVP-based HR), from now on referred to as True HR. To this end, first, we performed a Spearman correlation between True HR and rPPG HR.

As a second step, we tested whether the rPPG HR differed from the True HR using estimation statistics (based on the median difference (Mediff), given that the data were non-normally distributed) to estimate differences across measures analysis (bootstrapped Spearman correlation and estimation statistics) on the averaged HR scores.

### Study 2: Online validation

#### Participants

Eighteen participants took part in the study. One participant was removed from further analysis due to the extremely poor quality of the video recordings, leaving a total of 17 participants and 204 videos in the final dataset (11 females, six males, M_age_ = 33.11, SD = 14.34). Given that in Study 1 we applied rPPG extraction to all videos in the CohFace database, in Study 2 we aimed to obtain a dataset of a size comparable to the CohFace database. All participants provided written informed consent before participation. The study was approved by the Department of Psychology Ethics Committee at Royal Holloway University of London.

#### Procedure, video collection, and conversion

Participants were invited to perform an online experiment hosted on Gorilla Platform (https://gorilla.sc/). After signing the online consent forms, participants were instructed to download a validated (Losa-Iglesias, Becerro-de-Bengoa-Vallejo et al. 2016) mobile application to record their finger pulse heart rate (Heart Rate Plus App, version downloaded 07/2020 – https://play.google.com/store/apps/details?id=com.dungelin.heartrate). This application is used as a benchmark for the actual number of heartbeats (i.e., App HR). Participants were also instructed to activate the webcam and to i) sit in a well-illuminated room with natural light, ii) sit as still as possible during the recordings, iii) face the camera and sit close to the camera, iv) avoid any shadows on the face, v) avoid covering any part of the face (e.g., by wearing a mask, by hair or touching the face).

After the instruction, we used Gorilla’s beta video recording zone functionality to activate the webcam of the participants and to record videos of their faces. We recorded three videos with different durations (25, 35, and 45 s long) for each participant’s session. Participants were informed when the video recording started with an audio cue (i.e., a 200-ms beep). During the recording, they were instructed to look at a fixation cross displayed on a screen. Immediately after each recording interval, participants were instructed to measure their heart rate via the mobile phone application and to manually write in a text field the actual heart rate, which served as a control measure for the rPPG HR estimate. Participants were asked to repeat the experiment approximately seven times on separate days with at least a day in between, resulting in multiple video recordings for each participant. A total of 213 online video recordings were collected. The number of videos varied per participant as some did not complete the total of eight requested sessions. As such, some participated once (thus contributing with three videos), while others participated up to eight times (thus contributing with 24 videos). A total of 71 sessions were concluded and each participant finished 3.9 times (SD = 2.6) on average.

Heart rate measured via mobile application (App HR) immediately after each video recording provided a control measure for the rPPG HR estimate. We selected a mobile application with cross-platform availability, high reliability, high concurrent validity, and high consistency compared to a finger pulse oximeter (intraclass correlation (ICC), used to determine reliability between trials when using each system, pulse oximeter and App; ICC > 0.93; coefficients of variation of method errors (CVME), calculated for the absolute comparison of parameters; CVME = 1.66−4.06% (Losa-Iglesias, Becerro-de-Bengoa-Vallejo et al. 2016).

We converted the videos (resolution: 640 by 480 pixels) from Gorilla native video settings VP80 or VP90 codec and webm format to .mp4. For the conversion, we used HandBrake (https://handbrake.fr/) with the following parameters: Production Max, Quality Lossless (Placebo). All other pre-set parameters were left as they were. These conversion parameters created best-quality videos that allowed accurate frame-by-frame processing in MATLAB for the rPPG extraction.

#### Analysis

##### Data cleaning

As a first step, we removed videos for which the App HR control measure was missing. This could happen either because participants did not correctly input the App HR values (seven videos removed), or because of a system failure which resulted in the behavioral data not being uploaded to the Gorilla platform (three videos removed).

**Fig. 2:**
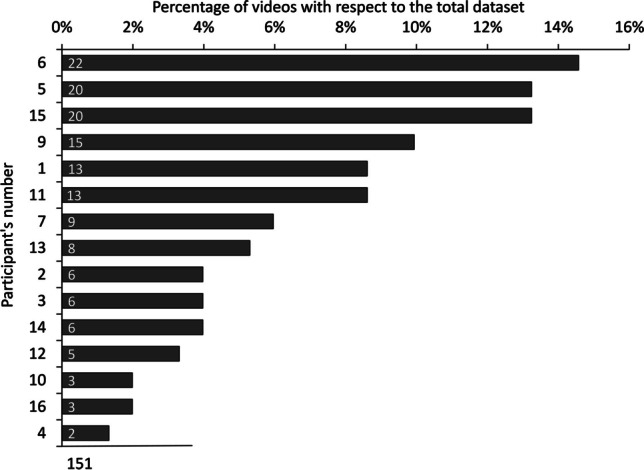
On the *x*-axis, the percentage of videos per participant, on the *y*-axis, the total number of videos in the dataset after the data cleaning procedure. The *labels inside the bars* show the total number of videos per participant (these were collected in different sessions spanning several days), and also the sum of all videos

Some videos could not be processed because the computer vision toolbox could not detect a face. As a result, for these videos, we could not compute the rPPG HR and they were removed from further analysis (five videos were removed). We then removed all videos with a frame rate lower than 20 frames per second (FPS; 24 videos removed). Thirdly, we removed all videos for which the HR as measured either with the mobile app or with the rPPG was lower than 50 or higher than 120 beats per minute (BPM), assuming that the parameters extraction failed (six videos were removed because the App HR was not in this range). Finally, we used the r *boxplot* function to detect and remove outliers, defined as the values outside the interquartile range, both for the App HR (two videos removed) and rPPG HR (six videos removed). This process left us with a total of 151 videos from 15 different participants (nine females, six males) for the final analysis (see Fig. 2).

##### rPPG extraction

Analyses of the videos were conducted in the same way as for Study 1.

##### Accuracy

First, we checked whether our variables of interest were normally distributed in the same fashion as for Study 1 by performing two separate Shapiro–Wilk tests of normality, one for App HR and one for rPPG HR. The analysis showed that data were normally distributed (see results), therefore we used parametric tests.

In order to test the accuracy of rPPG on our wild video dataset, we compared the rPPG HR to the App HR by performing a Pearson correlation.

Second, we calculated to what degree the rPPG HR differed from the App HR. To this end, we used estimation statistics based on confidence intervals (CIs) and represented with Cumming estimation plots (Cumming 2014, Ho, Tumkaya et al. 2019). We used the mean difference for two comparisons, shown with Cumming estimation plots. These plots show the raw data for each condition and the paired difference with 95% bias-corrected accelerated confidence interval based on 5000 bootstrap samples. Paired differences across measures were estimated based on mean difference (M_diff_), given that the data were normally distributed. Inference was based on the inspection of the estimated difference across conditions and the precision of such estimate (i.e., length of the CI): in accordance with previous works using this approach (Benassi, Frattini et al. 2021). CIs fully overlapping with 0 were interpreted as indicative of no evidence of difference between measures; CIs not overlapping with 0 were interpreted as indicative of weak, moderate, or strong evidence of difference between measures based on the size of the estimated difference and its precision, as the longer the CI, the weaker evidence there is (Cumming 2014, Calin-Jageman and Cumming 2019).

These statistics were computed using the web application available at: https://www.estimationstats.com/. Previous studies showed that the accuracy of the HR as recorded via rPPG also depends on the duration of the video analyzed (van der Kooij and Naber 2019). In that work, as more frames were added to the rPPG analysis, the more the correlation between the rPPG HR and the ground truth increased. In the present work, each experimental session comprised three video recordings: 25s, 35s, and 45s, hence, each video is composed of a different number of frames (the shorter the duration, the smaller number of frames). Thus, one could speculate that longer videos would provide more reliable estimates. Therefore, to compensate for possible fluctuation in the shorter videos collected, we averaged the HR calculated from the three videos aiming at increasing the correlation between the App HR and the rPPG HR. To test whether this is the case, we averaged the HR per each experimental session. Then, we repeated the previous analysis (bootstrapped Spearman correlation and estimation statistics) on the averaged HR scores.

## Results

### Study 1: CohFace dataset

#### Accuracy

**Fig. 3:**
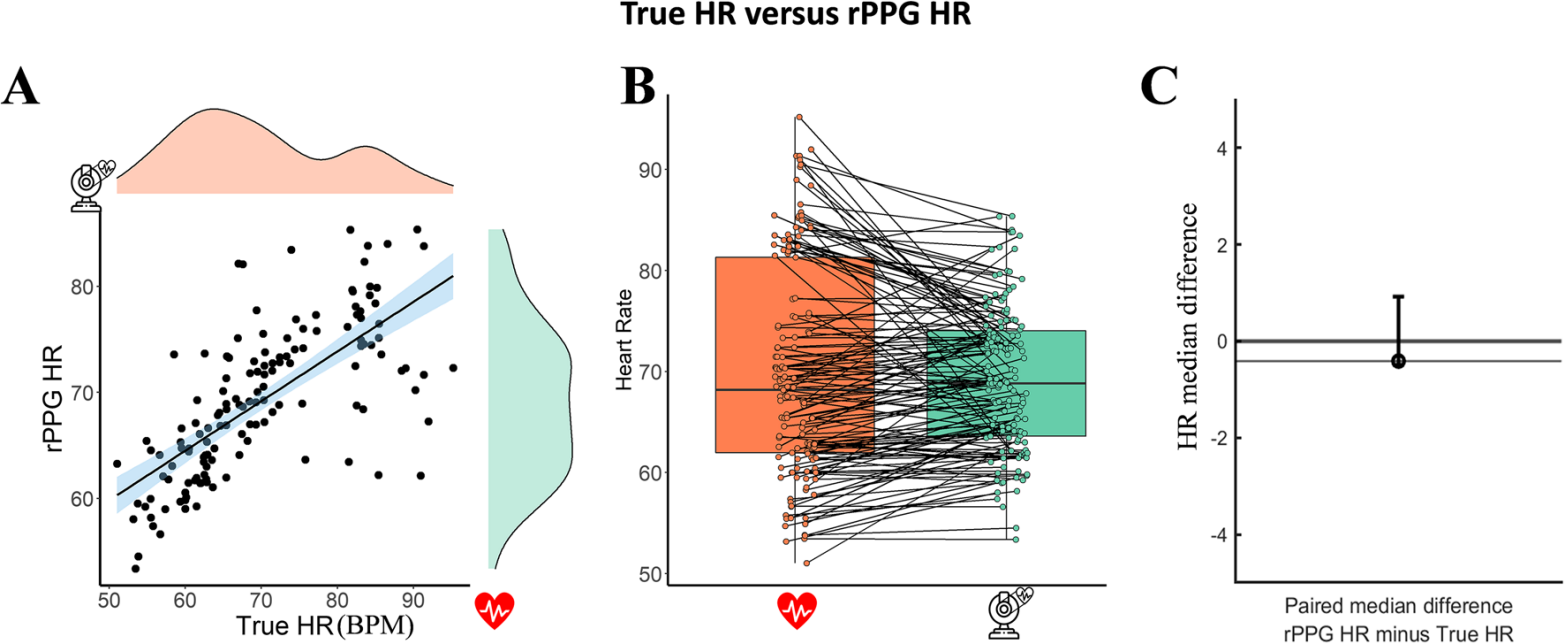
In the figure, *hearts* represent the true HR; *webcams* represent the rPPG HR. **A** The relation between the True HR and the rPPG HR; **B** boxplots for each condition with each paired set of observations connected by a line; **C** the paired median difference between the True HR and rPPG HR

Visual inspection of Q-Q plots and distribution plots showed that the data were not normally distributed. This was further confirmed by the Shapiro–Wilk tests of normality (True HR, *p* < 0.001, rPPG HR, *p* = 0.09). A bootstrapped Spearman correlation revealed a strong, positive correlation between True HR (IQR = 19.35, SD = 10.67) and rPPG HR (IQR = 10.43, SD = 7.12), which was statistically significant (see Fig. 3; r_s_ = 0.752, BCa 95% CI [– 0.642, 0.829], *p* < 0.001, see Fig. 3A). The paired median difference between True HR and rPPG HR is – 0.412 [95.0% CI – 0.335, 0.917]. This indicates that there was only a small difference between the real heart rate and heart rate scores extracted with the rPPG algorithm (see Fig. 3B, C).

**Fig. 4:**
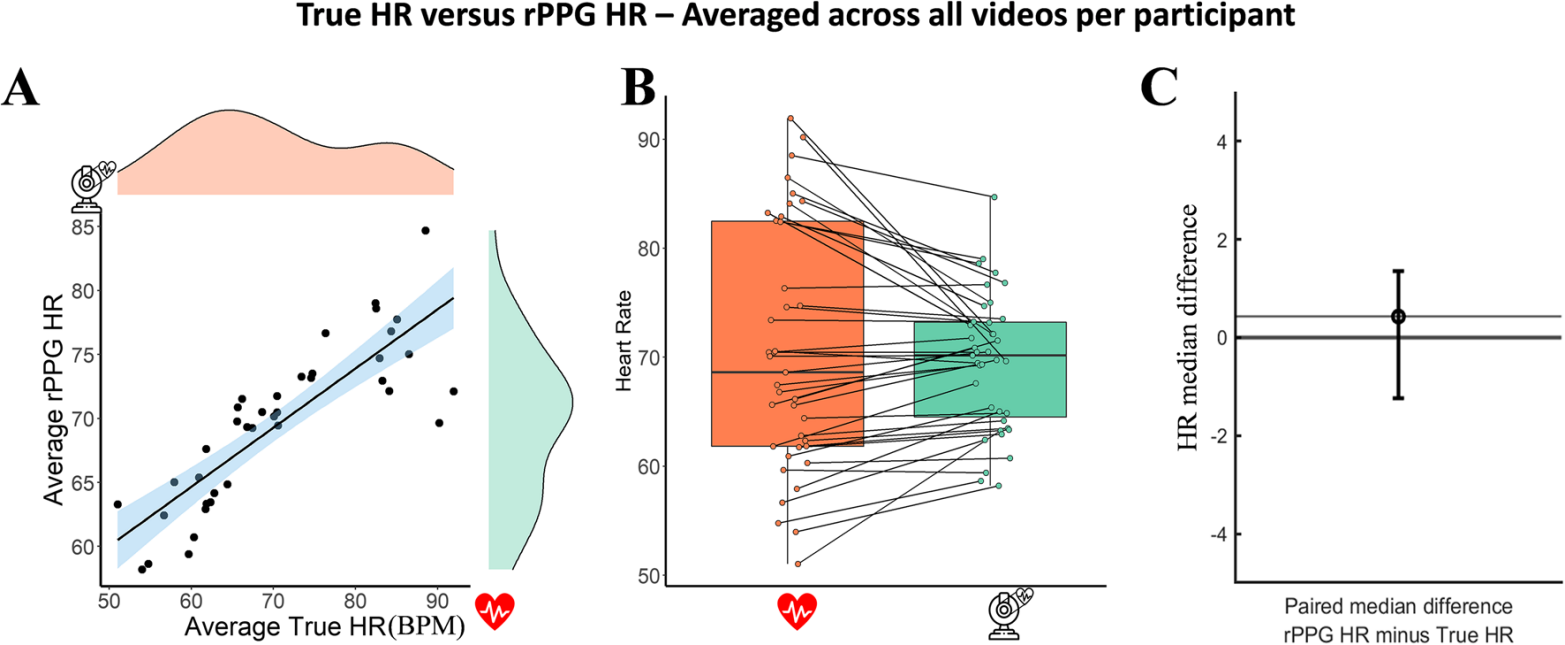
**A** The relation between the True HR averaged per participant and the rPPG HR; **B** boxplots for each condition with each paired set of observations connected by a line; **C** the paired median difference between the True HR and rPPG HR averaged per participant

The bootstrapped Spearman correlation performed on the HR averaged across all videos per participant (*N* participants = 39, average videos per participant = 3.6, SD = 1.1) revealed a strong, positive correlation between Mean True HR (mean = 70.59, SD = 11.17) and mean rPPG HR (mean = 69.56, SD = 6.15), which was statistically significant (see Fig. 4A, B); r_s_ = 0.873, BCa 95% CI [0.713, 0.935], *p* < 0.001). This indicates that recording multiple videos of the same participant substantially improves rPPG’s convergence.

The paired median difference between the average True HR (IQR = 20.63) and average rPPG HR (IQR = 8.70) is 0.429 [95.0% CI – 1.23, 1.36] (see Fig. 4C). In this case, averaging the videos did not improve the convergence of the rPPG as compared to the paired median HR difference of the non-averaged videos. Nonetheless, in both cases, the difference between the true HR and the rPPG HR scores was small.

### Study 2: online validation

#### Accuracy

Visual inspection of Q-Q plots and distribution plots showed that the data were normally distributed. This was further confirmed by the Shapiro–Wilk tests of normality (APP HR, *p* = 0.72, and rPPG HR, *p* = 0.15).

**Fig. 5:**
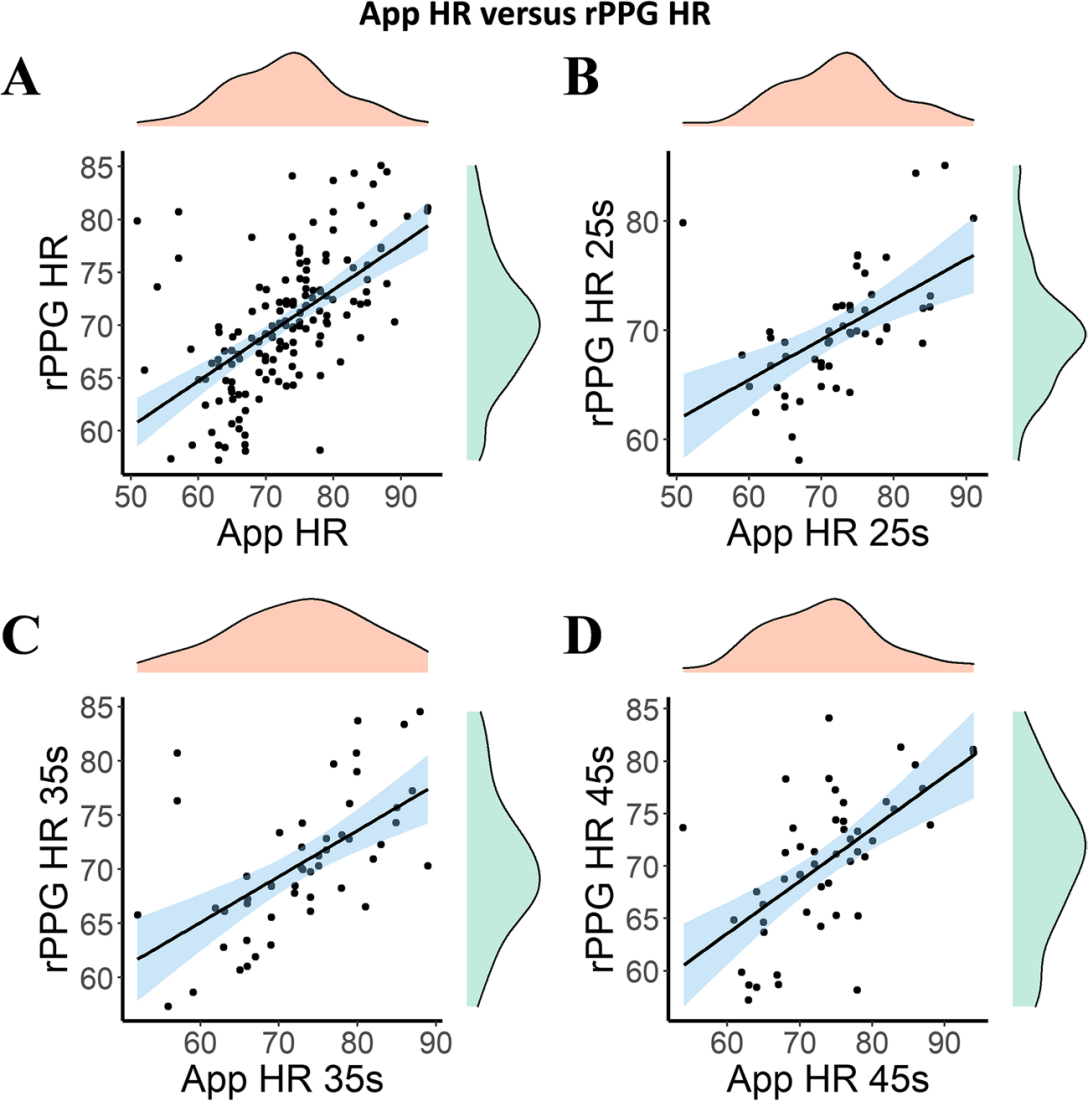
Relationship between the HR as measured with the Phone App and the HR calculated with the rPPG across all experimental sessions and all conditions (25, 35, and 45 s; for the analyses on the individual conditions see Supplementary Materials). **A** The relation between the two variables in all different timing conditions. **B**, **C**, and **D** The same relationship but for each condition separately (25, 35, and 45 s, respectively)

A bootstrapped Pearson correlation revealed a strong, positive correlation between App HR (across all sessions and conditions, 25, 35, and 45 s, mean = 72.83, SD = 8.34) and rPPG HR (mean = 70.27, SD = 6.22), which was statistically significant (*r* = 0.578, BCa 95% CI [0.386, 0.696], *p* < 0.001, see Fig. 5. For the analyses on the individual conditions, see Supplementary Material).

**Fig. 6:**
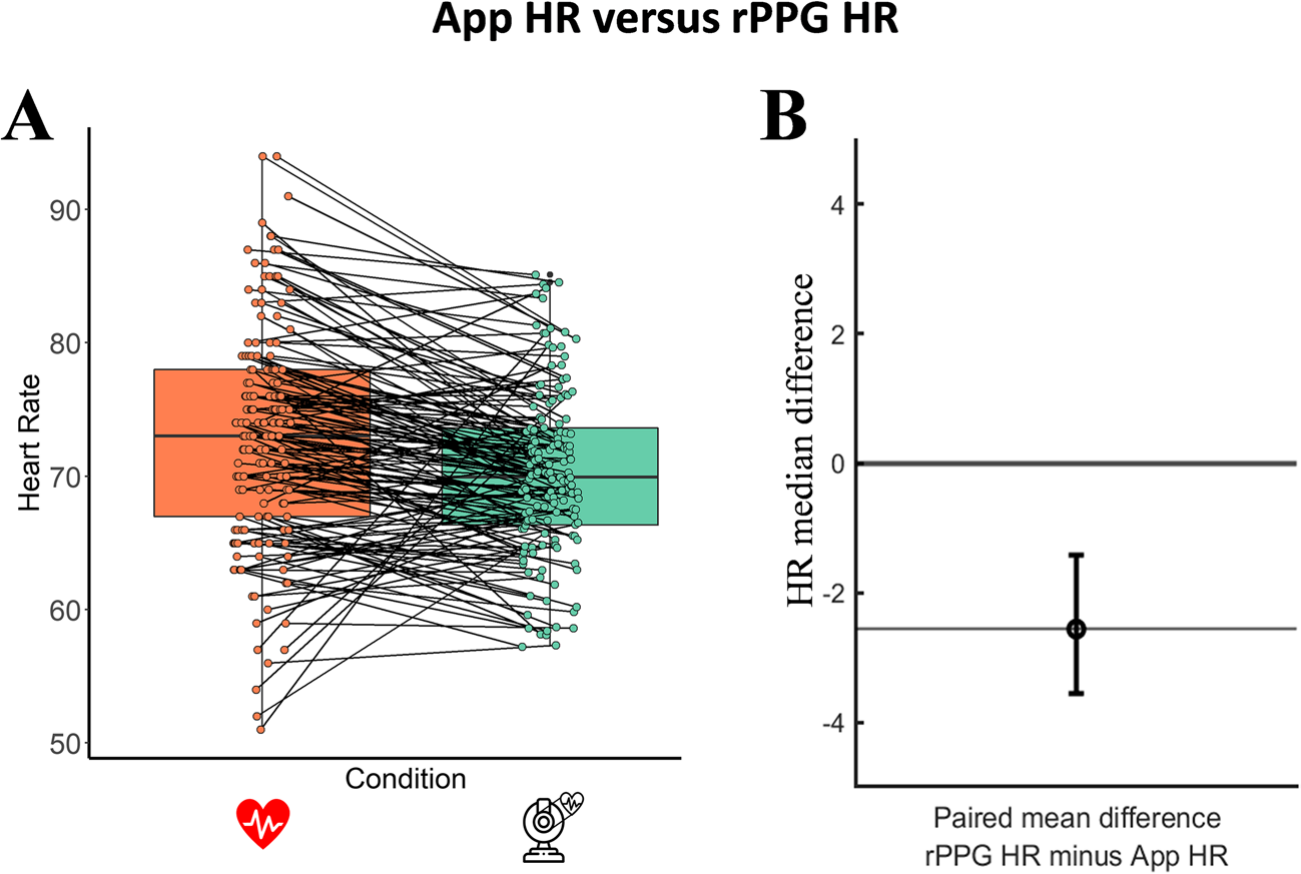
The paired median difference between App HR and rPPG HR with a Gardner–Altman estimation plot. The *left figure* shows boxplots for each condition and each paired set of observations is connected by a line. The *right figure* shows the paired mean difference between the two conditions: the median difference is depicted as a *dot*; the 95% confidence interval is indicated by the ends of the *vertical error bar*

When pooling all duration conditions, the paired mean difference between App HR and rPPG HR was – 2.56 (mean absolute error (MAE) = 5.50, [95.0% CI – 3.56, – 1.41], see Fig. 6A, B). This indicates that there was evidence of a moderate difference between heart rate scores as measured with the App and HR scores extracted with the rPPG algorithm. This difference means that rPPG underestimates HR with respect to the App HR, however, this difference is relatively small (5.56 heartbeats).

**Fig. 7:**
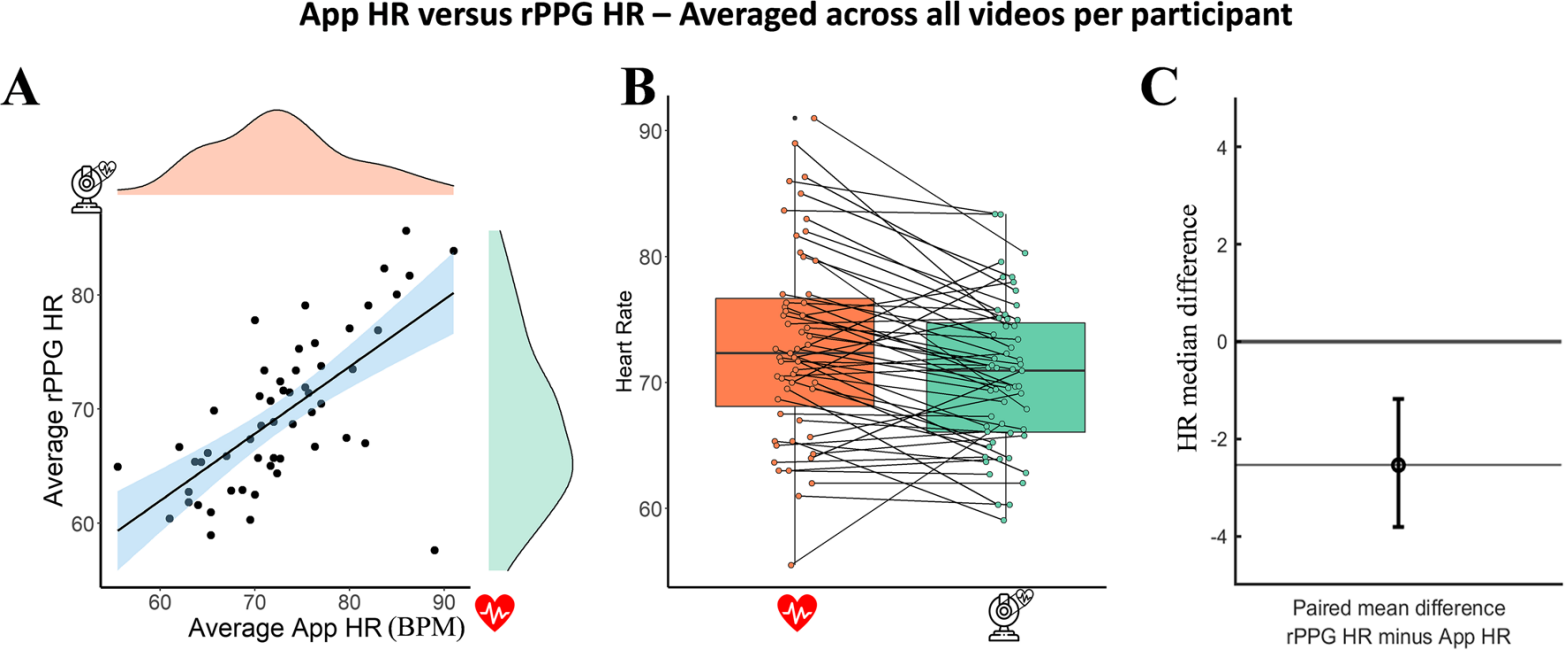
**A** The relation between the HR averaged per condition (25, 35, 45 s) and per session, as measured with the Phone App and the rPPG HR; **B** boxplots for each condition with each paired set of observations connected by a line; **C** the paired mean difference between App HR and rPPG HR averaged per session

Next, we checked whether averaging the HR calculated across the three duration conditions (25, 35, 45 s), per session (*N* = 55), would increase the correlation between the App HR and the rPPG HR. The bootstrapped Pearson correlation revealed a strong, positive correlation between Mean App HR (mean = 72.98, SD = 7.55) and mean rPPG HR (mean = 70.44, SD = 5.85), which was statistically significant (see Fig. 7A; *r*_s_ = 0.752, BCa 95% CI [– 0.610, 0.838], *p* < 0.001). Notably, the correlation coefficient is higher than the one for the non-averaged conditions, which seems to support the prediction that combining the rPPG HR for the three conditions would increase the accuracy of the estimated HR. However, the paired mean difference between the average App HR and average rPPG HR was – 2.54, MAE = 4.34, [95.0% CI – 3.81, – 1.18], indicating no improvement as compared to the – 2.56 mean difference for the non-averaged conditions (see Fig. 7B and  C).

## Discussion

The aim of this study was to demonstrate the functionality of a newly developed rPPG extraction algorithm to measure the heart rate via webcam, with a particular interest in its applicability to online studies.

We have conducted two experiments to demonstrate: (1) how the algorithm performed on an already validated dataset (CohFace) used for rPPG testing (Heusch et al., 2017), and (2) how the algorithm performed on videos recorded via an online platform, with little control over the environmental context and hardware setup of the participants being recorded, compared to a validated mobile heart rate application (Losa-Iglesias, Becerro-de-Bengoa-Vallejo et al. 2016).

Results from Study (1) show a high correlation between the rPPG heart rate and the true heart rate (*r* > 0.75), and no evidence of a difference between the two measures. Results from study (2) show that the HR extracted with our rPPG algorithm correlates (*r* = 0.58) with the HR recorded via a validated mobile application. However, there was also evidence of a difference between the two measures, with the rPPG underestimating the HR, but the average difference was relatively small (2.56 heartbeats).

The significance of our study lies in its ability to measure heart rate accurately through remote photoplethysmography (rPPG) in uncontrolled, online environments. In fact, to the best of our knowledge, this is the first study that tested the accuracy of a rPPG algorithm on videos collected in the wild, that is, with no control over several important variables, such as the webcam used by the participants, the luminosity of the room, the distance from the screen and participant motion, to mention a few. Despite this lack of control, the rPPG algorithm showed a high level of accuracy, making it a promising tool for remotely recording physiological measures. Our results have immediate and far-reaching implications for psychological and physiological research conducted online, a modality that has gained particular importance in the past years and due to the push for more inclusive and diverse research samples, which can be only reached via online sampling methods. The application of our rPPG online methodology in Ruisch, Mohr et al. (2023) explored the connection between inner bodily perception (i.e., interoception) and political ideology, underscoring the utility of our methodology in diverse fields. By offering a method to accurately measure heart rate online, our study expands the possibilities for researching physiological correlates of cognitive, social, and political phenomena in large and diverse samples. Among other things, this opens the possibility of expanding the field of interoceptive research, which has traditionally been confined to laboratory settings. By utilizing our rPPG methodology, researchers can explore various pertinent questions using large samples collected online, ranging from understanding the emotional states of other people (Arslanova, Galvez-Pol et al. 2022) to deployments of large online interoceptive assessments in healthy (Legrand, Nikolova et al. 2022)  and clinical populations (Di Lernia et al., 2019).

As noted by Rouast, Adam et al. (2018), rPPG methods have evolved significantly in the last decade, providing a range of modular approaches. Our work adds to this by offering a novel methodology designed specifically for uncontrolled, online environments. This makes our experimental pipeline particularly adaptable for researchers who need reliable rPPG data in diverse contexts. Moreover, our methodology addresses some of the common rPPG issues by delivering highly accurate heart rate measurements even when the recording conditions are less than ideal, partially tackling one of the critical barriers in achieving Sinhal's objective AAA (anyone, anywhere, and anytime) for vital sign detection (Sinhal et al., 2020), thus enabling broader and more inclusive research.

However, our work presents different limitations. An important aspect that can be observed from our data is that there is some degree of variation in the accuracy of the rPPG. Several factors may account for this variation, and it is reasonable to speculate that the most important is the overall quality of the video (e.g., frame rate, illumination, resolution, image background, and distance to camera). In this regard, as the resolution (and frame rate) cannot be optimized post hoc, any artificial spatiotemporal up sampling would add data points that reflect average or interpolated values of the original data. Therefore, optimization can only be accomplished before data collection (e.g., see Sinhal et al. (2020)), for example by instructing participants to use good lighting conditions to maximize framerate. Current online platforms for webcam-based video collection further limit screen resolutions, although it is currently unclear whether and how resolution may influence the rPPG’s signal-to-noise ratios. Notwithstanding, it is known that uneven illumination and oversaturation from a light source orthogonal to the participant’s face and the fluctuating light of the monitor reflection increase movement artefacts and reduce the performance of rPPG algorithms (e.g., Moço, Stuijk et al. (2016), Gudi, Bittner et al. (2020).

Another important factor is the level of physiological arousal when starting the experiment. It is likely that the algorithm’s performance is lower with high HR variability (van der Kooij and Naber 2019) because it is harder to accurately measure an unstable HR. Therefore, if participants engaged in an activity that increased their HR before taking part in the experiment, it could potentially decrease the accuracy of the rPPG algorithm. This observation is supported by visual inspection of the data (see Fig. 4A, B), which indicates higher estimation errors for lower and higher heart rates (particularly below 60 and above 90). Consequently, experimenters should take extra care when using rPPG in online experiments. It is particularly important to provide participants with clear instructions to minimize such artefacts and increase the quality of the video recordings and, therefore, the performance of the rPPG algorithm. In this regard, the development of a web-based feedback procedure on the positioning and illumination of the participants’ faces would be a welcome feature in future online experiments. Meanwhile, this evidence suggests that caution is particularly warranted when interpreting low and high estimated heart rates (< 60 & > 90) as our experiment suggests that these are more likely to be biased. For these estimated values, it is recommended to visually inspect the video recordings to check their quality and, also, to examine the power spectrum and the signal-to-noise ratio measure outputs provided by the algorithm to evaluate the plausibility of the estimated HR. Moreover, it must be noted that previous studies showed that rPPG performance drops significantly with darker skin tones (for a review, see Nowara et al. (2020)). As the CohFace dataset contains not enough diversity in the subject’s skin tone, a limitation of the present study is that we could not compare the accuracy of the algorithm on brighter versus darker skin tones. This limits the applicability of this tool for online testing, although it is important to mention that recent developments may help to mitigate bias in rPPG (Ba et al., 2021). Future studies should particularly aim at testing and correcting for differences in the accuracy of the algorithm on different skin tones.

In conclusion, taking these limitations into account, we have shown that our rPPG algorithm is accurate both when tested on validated videos recorded in a lab and when tested on videos recorded via an online platform, with no control over the recording hardware and the environmental conditions. This is beneficial, as it gives researchers another tool that they can use for recording and analyzing physiological parameters remotely, with inexpensive and widely available tools.

## Data Availability

The data that support the findings of this study are openly available in OSF at https://osf.io/9eymv/

## Code Availability (software application or custom code)

rPPG code is available here: https://github.com/marnixnaber/rPPG/tree/into-the-wild
